# Editorial: Digital health equity

**DOI:** 10.3389/fdgth.2023.1184847

**Published:** 2023-05-02

**Authors:** Hannah Crowe-Cumella, Joanne Nicholson, Adrian Aguilera, Terika McCall, Karen L. Fortuna

**Affiliations:** ^1^ Department of Social Work, Salem State University, Salem, MA, United States; ^2^ Heller School for Social Policy and Management, Brandeis University, Waltham, MA, United States; ^3^ UC Berkley Social Welfare, University of California, Berkeley, CA, United States; ^4^ UCSF Department of Psychiatry and Behavioral Sciences, UCSF Medical Center, San Francisco, CA, United States; ^5^ Division of Health Informatics, Department of Biostatistics, Yale School of Public Health, New Haven, CT, United States; ^6^ Section of Biomedical Informatics and Data Science, Yale School of Medicine, New Haven, CT, United States; ^7^ Center for Interdisciplinary Research on AIDS (CIRA), Yale School of Public Health, New Haven, CT, United States; ^8^ Geisel School of Medicine Geisel School of Medicine, Dartmouth College, Lebanon, NH, United States

**Keywords:** digital health, digital health intervention, health technologies, healthcare, healthcare interventions, health equity, digital health equity

**Editorial on the Research Topic** Digital health equity

Digital health technologies such as smartphone apps and remote monitoring present a promising path for intervention delivery; however, equal opportunities to engage in these technologies are a challenge in science presently. Some factors contributing to these inequalities include inaccessibility, exclusivity, redlining, and more. Innovations in digital health technologies must also be distributed equitably to avoid increasing existing disparities and improve overall population health and mental health. This editorial presents the state of the science on how digital health technologies can be leveraged to reach communities that are underserved and experience health disparities. Digital health is a growing phenomenon worldwide, and we must advance how all populations can leverage these technologies. This editorial reflects studies applying a social justice framework for digital health (Figueroa et al., 2022), including populations experiencing serious mental illness (Middle and Welch), homelessness (Lal et al.), and substance use (Claborn et al.). Studies also included those who are justice-involved (Tolou-Shams et al.), people of color, veterans, and youth and families. Below we summarize recent articles that focus on digital health equity.

Digital health technologies include telehealth, teletherapy, fitness apps, text messaging, computer programs, and smartphone apps. They are all promising approaches to intervention delivery for vulnerable and often underserved populations. Yet, many populations are left behind with the growing amount of digital health services. One article addresses the effect of digital health services on juveniles. When incarcerated, juveniles have little access to support services, including their family (Tolou-Shams et al.). Tolou-Shams et al. found that when telehealth and video conferencing services were made accessible, there was an increase in attendance to court hearings and telehealth interventions for youth and families. The authors also found that those involved with digital health interventions from the start of incarceration through community re-entry had higher levels of trust, enhanced engagement, and promoted the best youth outcomes.

Barriers to accessibility still exist for vulnerable populations despite government programs like Safelink and Assurance Wireless. Making digital health accessible is not always about using the most advanced technologies, but it's about using the best-fitted technology for the target population. Buda et al. state, “socio-economic and gender biases have been identified in healthcare systems, including digital divide problems caused by inequalities in access to digital services and lack of consideration for gender differences”.

Digital Redlining is a systematic process where underserved groups are deprived of equal access to digital tools, like availability to the internet (McCall et al.). Digital redlining creates inequities in access (McCall et al.) and may undermine opportunities to impact important health outcomes positively. Multiple articles in this series found that vulnerable populations were interested in digital health, but the systems in place were inaccessible. For example, Lal et al.'s article found that youth experiencing homelessness possessed the foundational skills, interests, and needs to participate in digital health interventions. However, these youth could not access digital health tools and technologies in their lived environment. Factors like internet access, access to phones/tablets, and data plans limit possibilities for homeless youth. Figueroa et al. (2021) found a similar phenomenon: Spanish-speaking women were highly interested in participating in fitness apps, yet the apps were unavailable in their primary language, making it user-friendly to only those well-versed in English.

Digital health continues to be ableist throughout our society, with limited to no accessibility options. Bunyi et al. provide several examples of ways to promote accessibility. For example, those who are deaf need in-app captions. Minimal apps offer this feature. Also, those on psychiatric medications could experience various side effects like tremors, memory impairments, and blurry vision. Most apps have text-heavy content, small print, and over-animation. This can be challenging, both visually and mentally, for many people. This article highlighted three areas of improvement that combat ableism: standards, research, and recognition. It explains that we must continue making strides to challenge and change digital health standards. Standards must be remade that is inclusive of all populations. Likewise, we must highlight the research being done into digital health delivery. Healthcare delivery must be measured with diverse populations. The only way we will change how digital health delivery is by creating new ways that are inclusive and equitable. Finally, we must transition our healthcare delivery to include user feedback. By recognizing user feedback, we create healthcare delivery methods that work for individuals vs. guessing what would work best for them.

Since the 1970s, manufacturing has shifted from engineer-centered to user-centered designs (Stiles-Shields et al.). Popular models for designing and deploying digital health technologies integrate human-centered design methodologies. These methodologies offer opportunities for users to contribute to developing technologies by co-designing and evaluating. Human-centered designs are an effective method for technology development and promote usability with the general population. However, some groups do not experience high levels of usability as they are commonly not involved in human-centered design or testing. For example, many technologies are developed for younger populations without input from these young individuals. Even adults aged 65+ increasingly use technologies that are not adequately designed for them. For example, smartwatches offer the opportunity to track steps and heart rates. They also monitor sleep, among many other things. As people age, a normal aging process is thinning the skin. Wearing a smartwatch that is not sensitive to thin skin may lead to cuts or bruises.

Incorporating a participatory human-centered design with users from populations who experience health disparities may help facilitate engagement and decrease unanticipated, technology-generated inequalities. Using feedback from intended users may minimize the gap between creating an intervention for someone and creating an intervention with someone. It allows us to work hand-in-hand with intended users to create user-friendly and relevant interventions that people want to engage with. This approach shifts the focus from the idea of “expert” or professionally driven design “for” the users to designing “with” users collaboratively (Porche, et al.) (1). Co-design can reduce potential harm or misuse by including people from vulnerable communities in decision-making (Porche et al.) (1). This allows local knowledge and expertise from marginalized voices to inform the development of more culturally relevant, trusted solutions.

Several studies show success with participatory human-centered interventions. Open2Chat is a stigma-free space for young people to articulate their concerns, share opinions and experiences with a peer, and discuss the idea of professional counseling or psychotherapy (Mittmann et al.). The results of this study showed value in co-development, like Open2Chat. Another study focused on a participatory human-centered design intervention for youth and families. This study pertained to mental health support, focusing on ways to boost self-determination. This intervention empowered participants to be informed and involved in their own treatment plans (Porche et al.). It also found that participatory human-centered design interventions created greater trust and safety among all parties involved. This fostered a more profound collaboration and community among users and researchers.

The results of these articles show a mix of benefits and concerns regarding digital health. While digital health can help reach diverse populations and create additional ways to access healthcare, it also raises concerns. Considerable research and work must be done to close the gap between healthcare delivery and issues of equity, accessibility, and inclusivity. It was shown to be successful and beneficial through participatory human-centered design, but most studies showed a lack of generalizability and raised questions of concern. Multiple forms of digital health have the potential to optimize healthcare. However, as we have new innovative ways to address healthcare, we must be conscious of populations we might be leaving behind due to inaccessibility and inequity. Throughout the editorial, we found evidence that most people will engage with digital healthcare if given access. Yet, we also found considerable concerns about how digital health is set up. We must make it a point to incorporate accessibility accommodations in every digital technology we utilize in the future. More studies should be done with these digital health interventions that eliminate the possibility of furthering inequalities. We look forward to building upon this set of studies and continuing research on digital health that centers on equity.
